# The Effect of Cyclooxygenase Inhibition on Tendon-Bone Healing in an *In Vitro* Coculture Model

**DOI:** 10.1155/2015/926369

**Published:** 2015-05-06

**Authors:** Tim Schwarting, Sebastian Pretzsch, Florian Debus, Steffen Ruchholtz, Philipp Lechler

**Affiliations:** Department of Trauma, Hand and Reconstructive Surgery, University Hospital Marburg, 35043 Marburg, Germany

## Abstract

The effects of cyclooxygenase (COX) inhibition following the reconstruction of the anterior cruciate ligament remain unclear. We examined the effects of selective COX-2 and nonselective COX inhibition on bone-tendon integration in an *in vitro* model. We measured the dose-dependent effects of ibuprofen and parecoxib on the viability of lipopolysaccharide- (LPS-) stimulated and unstimulated mouse MC3T3-E1 and 3T3 cells, the influence on gene expression at the osteoblast, interface, and fibroblast regions measured by quantitative PCR, and cellular outgrowth assessed on histological sections. Ibuprofen led to a dose-dependent suppression of MC3T3 cell viability, while parecoxib reduced the viability of 3T3 cultures. Exposure to ibuprofen significantly suppressed expression of *Alpl* (*P* < 0.01), *Bglap* (*P* < 0.001), and *Runx2* (*P* < 0.01), and although parecoxib reduced expression of *Alpl* (*P* < 0.001), *Fmod* (*P* < 0.001), and *Runx2* (*P* < 0.01), the expression of *Bglap* was increased (*P* < 0.01). Microscopic analysis showed a reduction in cellular outgrowth in LPS-stimulated cultures following exposure to ibuprofen and parecoxib. Nonselective COX inhibition and the specific inhibition of COX-2 led to region-specific reductions in markers of calcification and cell viability. We suggest further *in vitro* and *in vivo* studies examining the biologic and biomechanical effects of selective and nonselective COX inhibition.

## 1. Introduction

Rupture of the anterior cruciate ligament (ACL) of the knee is a common injury with a growing incidence in professional and recreational sportspersons [1, 2], reflected in the growing number of surgical ACL reconstructions performed each year [3]. Knee stability is generally restored by arthroscopic ligament reconstruction based on the transplantation of free autologous tendon grafts [4]. Despite being a well-established and highly standardized surgical procedure, approximately 10% of patients require operative revision because of graft failure and persistent joint instability [5, 6]. The interface between tendon and bone is of critical importance for the successful osseous integration of the transplant into the femoral and tibial tunnels [7]. However, the clinical issue of bone-tendon integration is not limited to ACL reconstruction but is a rather prominent challenge to orthopaedic surgeons treating ligament and tendon injuries at many other anatomical structures, including rotator cuff lesions, rupture of the distal tendon of the biceps brachii and scapholunar ligament, and the reconstruction of the medial patellofemoral ligament and lateral ankle joint stabilizators.

Conventional nonselective nonsteroidal anti-inflammatory drugs (NSAIDs) and selective cyclooxygenase- (COX-) 2 inhibitors are widely used following musculoskeletal trauma and as postoperative analgesics [8]. While the COX-1 isoform has been identified as a “housekeeper” enzyme that is constitutively expressed in almost all tissues, COX-2 is the product of an immediate-early gene that is rapidly inducible and tightly regulated [9]. The expression of COX-2 is highly restricted but is sharply upregulated during inflammatory processes. Both COX-1 and COX-2 are key enzymes in the synthesis of prostaglandins (PGs), which are important factors in bone metabolism and fracture healing [9]. Whereas COX-1 and COX-2 inhibitors reportedly impair bone formation, they both appear to enhance tendon-bone integration [8].

We examined the effects of nonselective COX inhibition and selective COX-2 inhibition on the interaction between osteoblasts and fibroblasts at the tendon-bone interface* in vitro*.

## 2. Materials and Methods

If not otherwise specified, reagents were sourced from Sigma-Aldrich (Taufkirchen, Germany) and consumables from Sarstedt (Nümbrecht, Germany). All experiments were performed in triplicate.

### 2.1. Cell Lines

Cells from the murine MC3T3-E1 (preosteoblast) and 3T3 (fibroblast) lines (DSMZ, Braunschweig, Germany) were cultivated at 37°C in a humidified atmosphere of air and 5% CO_2_ in Eagle's minimum essential medium, *α* modification (*α*MEM), and Dulbecco's modified essential medium (DMEM), respectively. Both media were supplemented with 10% fetal calf serum, 100 U/I penicillin, and 100 *μ*g/mL streptomycin. Experiments were performed at passages 7 to 9. MC3T3-E1 cells were adapted to DMEM 10 days before the initiation of the coculture.

### 2.2. MC3T3-E1/3T3 Coculture Model

**Figure 1:**
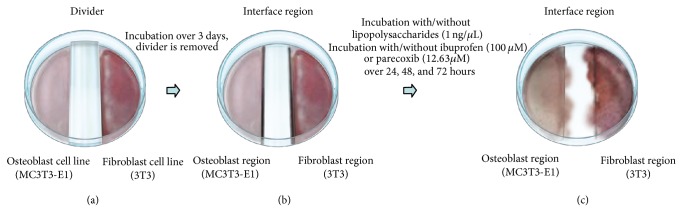
Illustration of the murine coculture model simulating bone-tendon integration* in vitro.*

**Figure 2:**
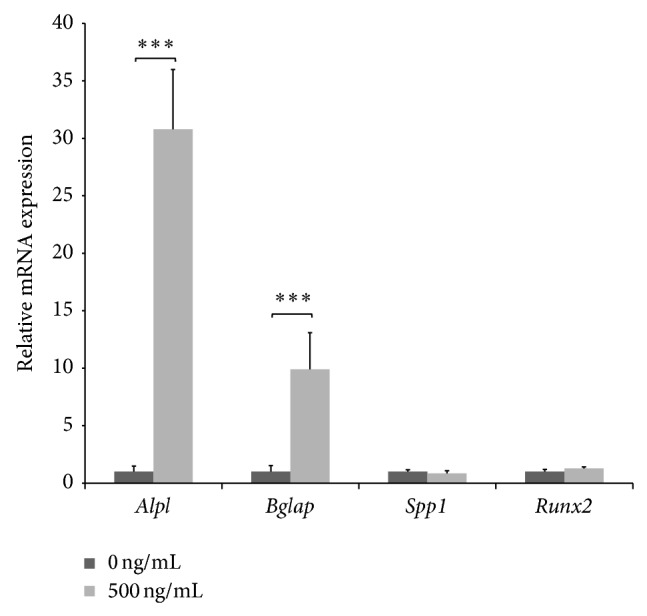
Stimulation of gene expression by recombinant BMP-2. Induction of target gene expression three days following the application of recombinant BMP-2 (500 ng) at the osteoblast region served as positive control. Gene expression was measured 3 days after the stimulation with BMP-2 (500 ng/mL) and normalized to housekeeping genes.* Alpl*: alkaline phosphatase;* Bglap*: bone gamma-carboxyglutamate protein; BMP-2: bone morphogenetic protein-2;* Runx2*: runt-related transcription factor 2. *P* values are provided as follows: ^∗^ *P* < 0.05, ^∗∗^ *P* < 0.01, and ^∗∗∗^ *P* < 0.001.

A murine coculture model providing osteoblast, interface, and fibroblast regions as described by Wang and colleagues [10] was used to study the effects of COX inhibition on bone-tendon healing* in vitro*. A sterile agarose divider (1.2 cm width) was fixed to the base of a plastic cell culture dish, and 1 × 10^6^ MC3T3-E1 cells were seeded on the left and 1 × 10^6^ 3T3 cells on the right of the divider (Figure 1). Cells allowed become adherent over 10 min; then the cocultures were covered with fully supplemented DMEM with 10 *μ*g/mL ascorbic acid and 1 mM glycerol-2-phosphate. After 3 days under standard culture conditions, the agarose divider was removed. Cultures were stimulated with lipopolysaccharide (LPS) at a concentration of 1 ng/*μ*L over 4 hours. Next, the supernatant was decanted and the nonselective COX inhibitor ibuprofen (Sigma-Aldrich, Steinheim, Germany) or the specific COX-2 inhibitor parecoxib (Pfizer, New York City, NY, USA) was added. At days 2 and 3, medium was carefully renewed. Medium was removed and cocultures were rinsed with ice-cold phosphate-buffered saline (PBS). A region-specific cell harvest was performed by applying 600 *μ*L Buffer RLT (lysis buffer in the RNeasy Kit, Qiagen, Hilden, Germany) to the upper border of the respective region, while the plate was held in a tilted position. For each area, lysed cells were removed from the plate using a cell scraper and collected in three separate tubes (osteoblast, interface, and fibroblast regions). Lysates were stored at −80°C. Cultures treated with dimethyl sulfoxide (DMSO, 520 *μ*M) and NaCl (36.02 *μ*M) served as negative experimental controls. As a positive control, the stimulatory effects of recombinant bone morphogenetic protein- (BMP-) 2 at 500 ng/mL (InductOs, Dibotermin Alfa, Pfizer, Berlin, Germany) were measured at day 3. The application of BMP-2 resulted in a significant regulation of the target genes at the osteoblast region (Figure 2).

### 2.3. Real-Time Quantitative Polymerase Chain Reaction (qPCR)

A RNeasy Mini Extraction Kit (Qiagen, Hilden, Germany) was used to extract the RNA. Complementary DNA (cDNA) synthesis was performed in a FlexCycler thermal cycler (Analytik-Jena, Jena, Germany) using the iScript cDNA synthesis kit (Bio-Rad, Munich, Germany) according to the manufacturer's instructions. Standards were prepared by a tenfold dilution series between 1 and 1 × 10^−5^. cDNA was stored at −20°C.

For qPCR, the SsoFast EvaGreen Supermix (Bio-Rad) and 1 *μ*g of cDNA were processed in a Bio-Rad C1000 ThermoCycler running the CFX96 Real-Time System. Cycling conditions were 30 sec at 95°C, 40 cycles of 5 sec at 95°C, and 10 sec at 60°C. Finally, a melt-curve analysis with 0.5°C increments every 5 sec from 65°C to 95°C was performed.

The target genes were* Alpl* (alkaline phosphatase),* Bglap* (bone gamma-carboxyglutamate (gla) protein or osteocalcin),* Fmod* (fibromodulin), and* Runx2* (runt-related transcription factor 2); reference genes were* Actb* (actin beta) and* Hprt* (hypoxanthine guanine phosphoribosyl transferase). Primers were obtained from Qiagen and Sigma-Aldrich (Table 1). The primer covered at least one exon-intron junction and the negative first-deviation plots of the melting curve revealed specificity. Target gene expression was assessed using CFX Manager 3.1 software (Bio-Rad) and normalized to the reference genes.

### 2.4. Real-Time Cell Viability Analysis

To measure the influence of ibuprofen and parecoxib on the viability of MC3T3-E1 and 3T3 cells, the xCELLigence Real-Time Cell Analyzer (Roche Applied Science, Mannheim, Germany) and RTCA 1.2.5 software were employed according to the manufacturer's instructions. Briefly, 5,000 cells were seeded per well and incubated for 24 h (3T3) or 48 h (MC3T3) under standard conditions. Next, the medium was changed and cells were exposed to final concentrations of ibuprofen or parecoxib of 0 *μ*M, 5 *μ*M, 10 *μ*M, 25 *μ*M, 50 *μ*M, or 100 *μ*M. Controls were treated with either NaCl (36.02 *μ*M) or DMSO (520 *μ*M). The experiment was performed over 100 h at 37°C in a humidified atmosphere of air and 5% CO_2_. The cell index was acquired automatically every 15 min.

### 2.5. Microscopic Analysis of Proliferation and Migration at the Interface Region

To measure proliferation and migration at the interface region, the established coculture model was performed on sterile object slides (Gerhard Menzel, Braunschweig, Germany) as outlined above. The divider was removed 24 h after seeding and cultures were stimulated with LPS (1 ng/*μ*L) over 4 h. Next, cells were exposed to ibuprofen (100 *μ*M) or parecoxib (12.63 *μ*M); DMSO (520 *μ*M) and NaCl (36.02 *μ*M) treated cultures served as controls. The experiment was terminated at day 0, 3, or 7. Object slides were rinsed with PBS and cells were fixed for 15 min in 4% formalin. Next, slides were carefully washed with PBS, and a hematoxylin and eosin stain was performed. Slides were observed and photographed with a Leica DM LB microscope (Leica Mikrosysteme Vertrieb, Wetzlar, Germany). Quantification of combined migration and proliferation was performed by counting all visible cells in one-quarter of a visual field (at 20x magnification) of the osteoblast, interface, and fibroblast regions. For each experimental condition, slides of five independent cultures were analyzed.

### 2.6. Statistical Analysis

Statistical analysis was undertaken using SPSS for Mac, version 22 (SPSS, Chicago, IL, USA). All datasets were tested for normality with chi-square analysis. Groups were compared by employing one-way analysis of variance with Dunnett's multiple comparison. Statistical significance was set at *P* < 0.05. Graphs were plotted using Microsoft Excel for Mac, version 14.1.0 (Microsoft, Redmond, WA, USA).

## 3. Results and Discussion

### 3.1. Dose-Dependent Effects of Ibuprofen and Parecoxib on Cell Viability

**Figure 3:**
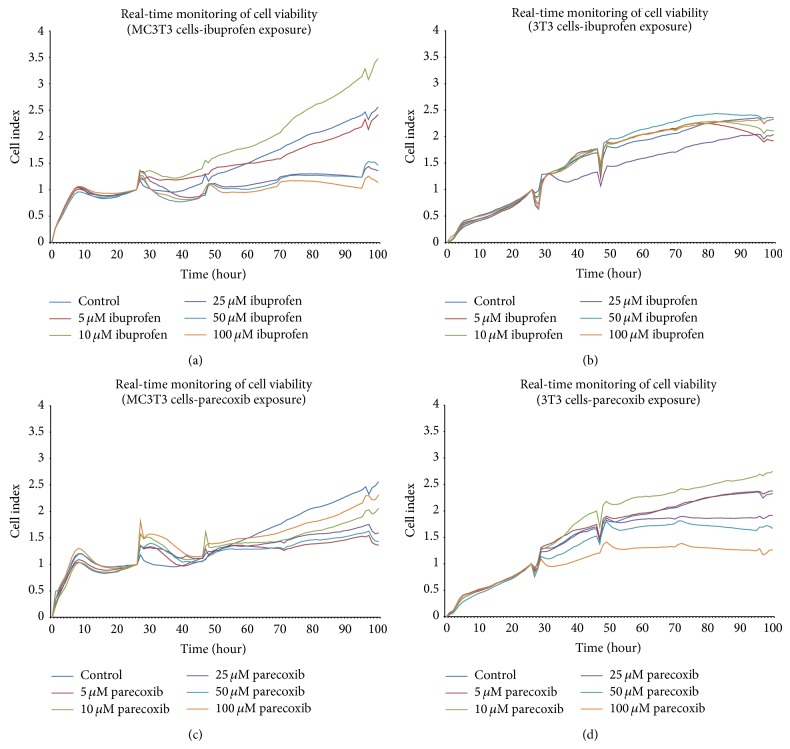
(a)–(d) Effects of ibuprofen and parecoxib on viability of cultured MC3T3-E1 (preosteoblast) and 3T3 (fibroblast) cells. Real-time cell viability was analyzed following the exposure to ibuprofen or parecoxib at concentrations of 0 *μ*M, 5 *μ*M, 10 *μ*M, 25 *μ*M, 50 *μ*M, or 100 *μ*M by xCELLigence over 100 h.

The dose-dependent effects of ibuprofen and parecoxib on the viability of cultured MC3T3-E1 and 3T3 cells are shown in Figure 2. Ibuprofen provoked a dose-dependent reduction in the viability of MC3T3 cells from 48 h onwards when compared with untreated controls (Figure 3(a)), but there was no apparent effect on 3T3 cells (Figure 3(b)). In contrast, parecoxib led to a significantly reduced cell viability of 3T3 cultures (Figure 3(d)), but no dose-dependent impairment of cell viability in MC3T3 cells (Figure 3(c)).

### 3.2. Effects of Ibuprofen and Parecoxib on Gene Expression at the Osteoblast, Interface, and Fibroblast Regions during Bone-Tendon Integration* In Vitro*

**Figure 4:**
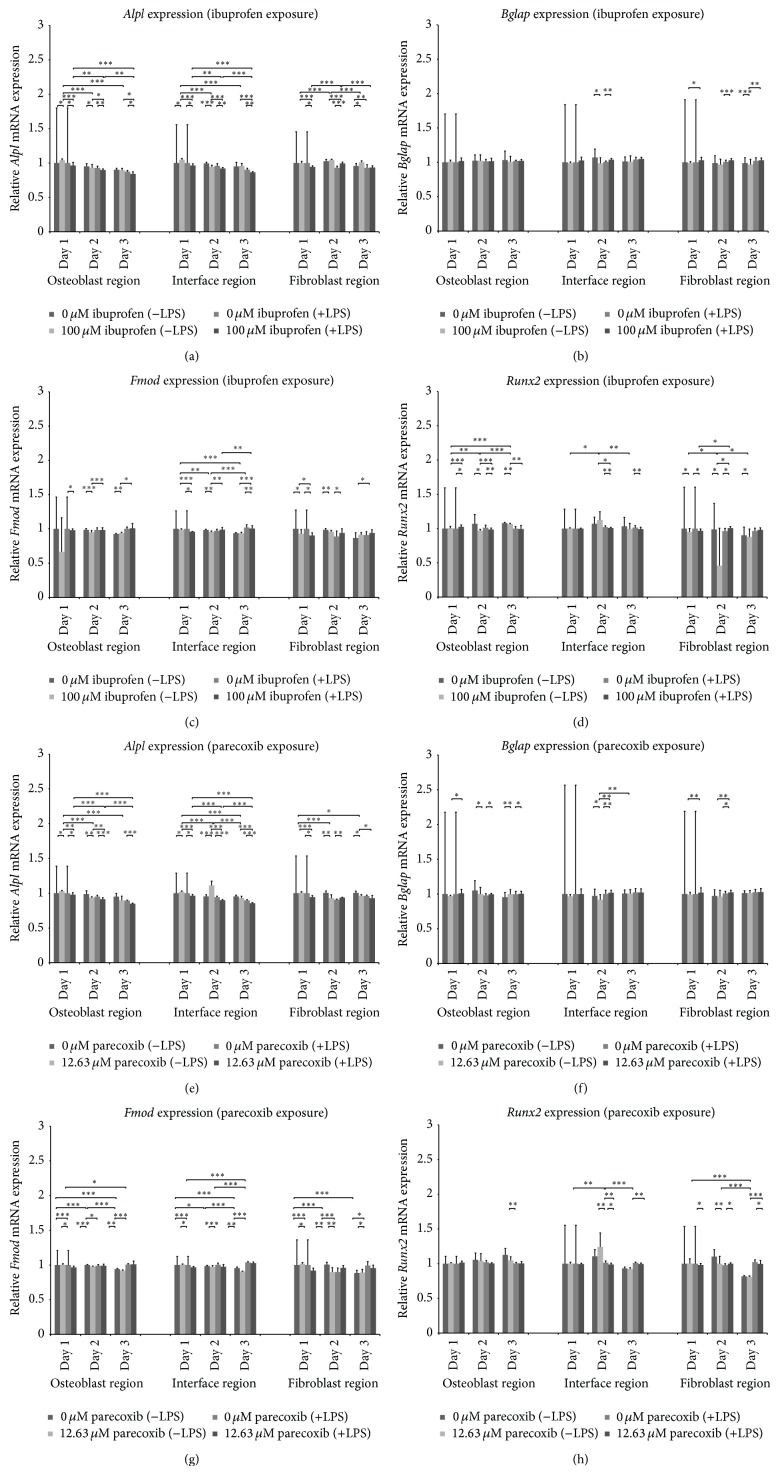
(a)–(h) Effects of ibuprofen and parecoxib on gene expression at the osteoblast, interface, and fibroblast regions during bone-tendon integration* in vitro*.* Alpl*: alkaline phosphatase;* Bglap*: bone gamma-carboxyglutamate protein;* Fmod*: fibromodulin; LPS: lipopolysaccharide;* Runx2*: runt-related transcription factor 2. *P* values are provided as follows: ^∗^ *P* < 0.05, ^∗∗^ *P* < 0.01, and ^∗∗∗^ *P* < 0.001.

The region-specific effects of ibuprofen and parecoxib treatment on the expression of* Alpl*,* Bglap*,* Fmod*, and* Runx2* in LPS-stimulated and unstimulated cultures were analyzed by qPCR at 24, 48, and 72 h. Unstimulated cocultures served as controls. Isolated LPS-exposure led to significantly decreased rates of* Alpl* expression at all regions (Figures 4(a) and 4(e)), while* Bglap*,* Fmod,* and* Runx2* showed no definite regulation by LPS. Ibuprofen treatment led to reduced* Alpl* expression at the osteoblast and interface regions of LPS-treated and -untreated cultures, while no distinct regulation was noted at the fibroblast region (Figure 4(a)). Parecoxib reduced* Alpl* expression in all three regions (Figure 4(e)).* Bglap* expression was significantly downregulated by ibuprofen in LPS-untreated cultures at the interface and fibroblast regions, while expression in LPS-stimulated cells was increased significantly by ibuprofen at the interface and fibroblast regions (Figure 4(b)). Although there were significant increases in* Bglap* expression in the LPS-stimulated cultures following parecoxib treatment, a nonsignificant trend towards a decrease was also noted in unstimulated cultures (Figure 4(f)). There was no consistent regulation of* Fmod* by ibuprofen (Figure 4(c)), while parecoxib resulted in decreased* Fmod* expression (Figure 4(g)).* Runx2* expression was downregulated by ibuprofen and parecoxib at all three regions (Figures 4(d) and 4(h)).

### 3.3. Effects of Ibuprofen and Parecoxib on the Microscopic Morphology and Outgrowth of the Interface Region during Bone-Tendon Integration* In Vitro*

**Figure 5:**
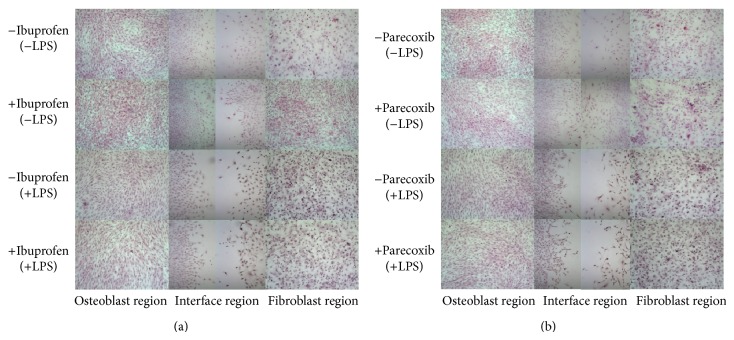
Microscopic morphology of the osteoblast, interface, and fibroblast regions during bone-tendon healing* in vitro*. Seven days following exposure to ibuprofen or parecoxib, murine cocultures were stained with hematoxylin and eosin, and representative regions were observed and photographed (20x magnification). *P* values are provided as follows: ^∗^ *P* < 0.05, ^∗∗^ *P* < 0.01, and ^∗∗∗^ *P* < 0.001.

**Figure 6:**
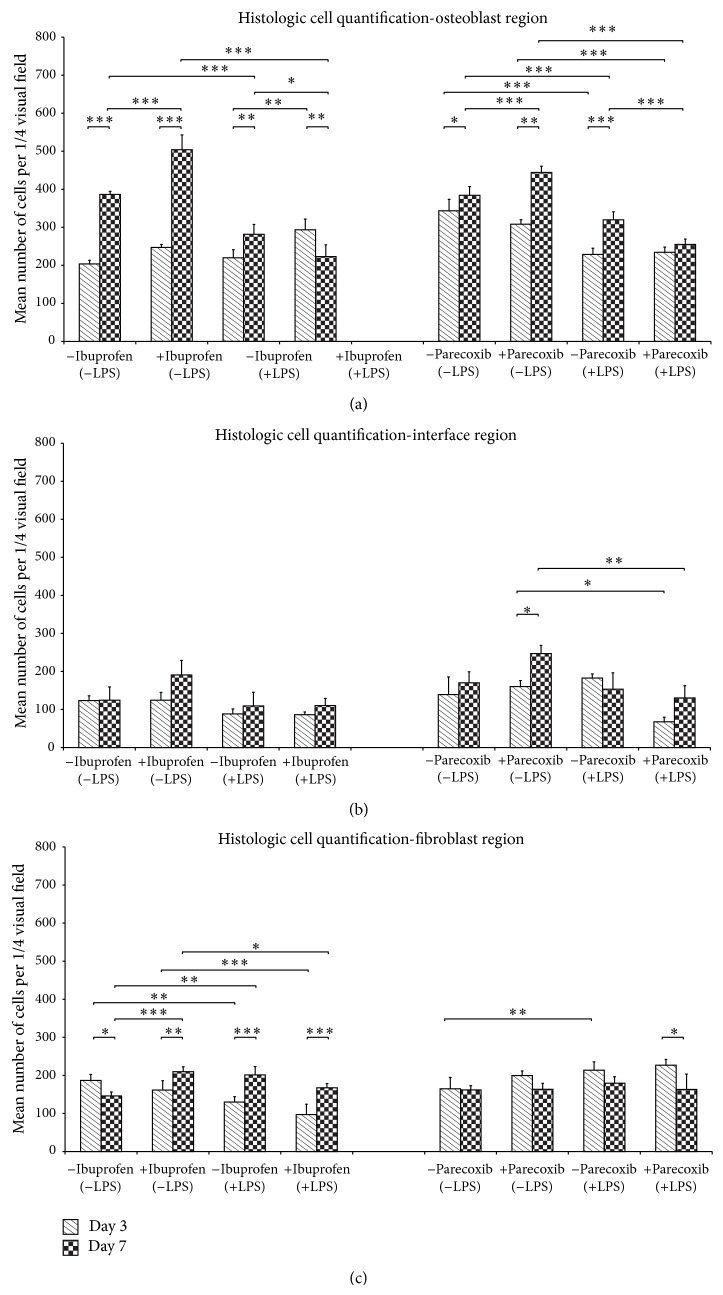
(a)–(c) Effects of ibuprofen and parecoxib on region-specific cellular outgrowth during bone-tendon healing* in vitro*. DMSO: dimethyl sulfoxide; LPS: lipopolysaccharide; NaCl: sodium chloride. *P* values are provided as follows: ^∗^ *P* < 0.05, ^∗∗^ *P* < 0.01, and ^∗∗∗^ *P* < 0.001.

Histological growth patterns of MC3T3-E1 and 3T3 cells at the osteoblast interface and fibroblast regions at day 7 are depicted in Figure 5. The quantitative analysis of cell outgrowth (combined proliferation and migration) revealed no inhibitory effects by ibuprofen in unstimulated cultures at the interface or fibroblast regions (Figure 6(a)), whereas the cell count was significantly increased in the osteoblast region following exposure to ibuprofen (*P* < 0.001, Figure 6(a)). However, in LPS-stimulated cocultures, ibuprofen treatment resulted in a significant decrease in the number of detectable cells at the osteoblast region (*P* < 0.05) and a trend towards reductions at the interface and fibroblast regions. These results were mirrored following exposure to parecoxib (Figure 6(b)), where unstimulated cultures showed increased cellular outgrowth at the osteoblast region, and inhibitory effects were noted at all three regions following LPS-stimulation.

### 3.4. Assessment of Findings

Ibuprofen, parecoxib, and other NSAIDs are a key part of many postoperative analgesic regimes following musculoskeletal surgery [11]. While the development of COX-2 specific drugs aimed to improve the efficacy and safety of NSIADs, their superiority over conventional nonselective COX inhibitors remains a matter of substantial controversy [12].

Induction of COX-2 and its biochemical product prostaglandin H2 is an important part of the healing process in musculoskeletal tissues [13], including bone [13] and tendon [14]. In musculoskeletal tissue, COX-2 is involved in a multitude of complex biological processes, including the recruitment and activity of proinflammatory cells and the formation and maturation of restored tissue [15]. We used a highly controlled* in vitro* model to investigate the influence of COX inhibition on bone-tendon integration. While we found marked suppression of markers of bone and noncalcified extracellular matrix formation following ibuprofen and parecoxib exposure, the published literature on the role of COX inhibition on bone-tendon healing remains controversial [16]. Several studies have reported beneficial effects of anti-inflammatory measures on tendon healing. Oak and colleagues showed that inhibition of 5-lipoxygenase, COX-1, and COX-2 led to improved tendon healing [17], and Krivic and colleagues reported that tendon healing was enhanced by an immunosuppressive protocol involving the peptide BPC 157 and systemic methylprednisolone* in vivo *[18]. Further evidence indicating the advantageous effects of limiting inflammation in injured tendons was provided by the study of McCarrel and colleagues, who reported that leukocyte-reduced platelet-rich plasma was superior to standard platelet-rich plasma in an* in vitro* model [19].

In contrast, Kocaoglu and colleagues reported that immunosuppression with mitomycin-C had no effect on the tensile load required to rupture repaired tendons, even though microscopic signs of inflammation were significantly decreased [20]. Although another group found that specific selective COX-2 inhibition with celecoxib did not influence tendon healing [21], it has been reported that systemic ibuprofen administered in the first postoperative week has detrimental effects on tendon healing in a rat supraspinatus tendon repair model (although ibuprofen administered only in the second postoperative week did not) [22].

The role of COX inhibition on bone formation and fracture healing has been studied extensively [23], but there is still significant controversy about the specific effects of selective COX-2 and nonselective COX inhibition. The majority of published studies reported reduced rates of callus formation and delayed fracture healing following treatment with selective* and* nonselective COX inhibitors [24–31]. Interestingly, Utvåg's group found that both selective COX-2 inhibition and nonselective COX inhibition administered for only the first 7 days after the fracture did not significantly influence bone regeneration and concluded that short-term treatment with an NSAID might not impair fracture healing [32].

While O'Connor and colleagues reported that treatment with the selective COX-2 inhibitor rofecoxib resulted in less effective fracture healing compared with ibuprofen [30], Gerstenfeld et al. found that parecoxib had only a minimal effect on healing, even at doses that are known to fully inhibit prostaglandin production [25]. The latter authors' conclusion that a selective COX-2 inhibitor has less effect on fracture repair compared with nonselective NSAIDs has been challenged, as the pharmacokinetic characteristics of the drugs used in the study can be unpredictable [33]. While we found some drug-specific differences, both selective and nonselective COX inhibition led to significant impairment of* in vitro* bone-tendon healing.

A relevant limitation of the majority of the previously published* in vitro* studies is the lack of an adequate proinflammatory stimulus in the experimental setup. For the present study LPS was administered to induce inflammatory signaling in both cell lines, thus simulating the early postoperative situation following ACL reconstruction. LPS has been reported to be a proinflammatory stimulus in MC3T3-E1 cells by Guo et al. [34]. The authors noted an LPS triggered activation of the JNK pathway, eventually leading to an inhibition of osteoblast differentiation and programmed cell death. In their classic paper, Arakawa et al. reported on the effects of LPS on the expression of the prostaglandin receptor gene EP4 in 3T3 cells [35], underlining the proinflammatory effect of LPS in this murine mesenchymal cell line.

### 3.5. Study Limitations

While using an established* in vitro* coculture model enabled highly standardized and reproducible experiments to be performed, the complexity of bone-tendon healing, including the involvement of other cell types in a complex three-dimensional extracellular matrix, could not be reproduced. Furthermore, no biomechanical influences on the model were assessed. Another potential limitation could be the use of established murine cell lines; however, the rationale for using murine cell lines instead of human cell cultures was primarily the excellent characterization of their molecular background by previous studies. Next, a wide range of molecular tools have been constructed for murine cells, enabling complex experimental approaches for future studies. Furthermore, MC3T3-E1 and 3T3 cells are globally available, thus improving the reproducibility of our data.

Next, we measured gene expression but did not assay protein concentrations. Furthermore, the expression analysis was limited to four genes serving as surrogate parameters of osteointegration (*Alpl*,* Bglap*, and* Runx2*) and extracellular matrix formation and maturation (*Fmod*). The role of* Alpl*,* Bglap*, and* Runx2* in osteoblast differentiation [36] has been studied extensively, and their importance for bone-tendon integration has been underlined previously [37, 38]. Fibromodulin (*Fmod*) has been identified to be a key participant in the organization of extracellular matrix by interacting with collagen fibrils and has been described as an important structural component of tendon and cartilage tissue [39]. However, the authors are aware that the analysis of the effects of the COX inhibition on noncalcified extracellular matrix formation and maturation is limited and should be further dissected by future studies employing long-term cultured tendon specimens [37] and animal models [7].

Another drawback of the study is the limitation on two COX inhibitors, that is, ibuprofen and parecoxib. However, both ibuprofen and parecoxib are widely used as anti-inflammatory drugs and have become the postoperative therapeutic standard in orthopedic practice. Thus, the present study aimed to transfer the clinical issue of a potential interference between ibuprofen or parecoxib and bone-tendon healing to the highly controlled experimental setup of a cell based* in vitro* study.

Finally, the induction or inhibition of COX-1 and COX-2 was not specifically measured.

## 4. Conclusions

Nonselective COX inhibition and the specific inhibition of COX-2 resulted in region-specific reductions in expression of markers of calcification and extracellular matrix synthesis* in vitro*. Furthermore, parameters indicative of cell viability, proliferation, and migration were suppressed by COX inhibition. As NSAIDs are in widespread clinical use to manage pain after ACL surgery, further* in vitro* and* in vivo* studies examining the biologic and biomechanical effects of COX inhibition are needed.

## Figures and Tables

**Table 1:** Primer sequences of target and reference genes.

Target gene	Product size (base pairs)	Annealing temperature (°C)	Sequence
*Actb *	200	66.5°C	Forward: 5′ CTCTGGCTCCTAGCACCATGAAGA 3′
		66.3°C	Reverse: 5′ GTAAAACGCAGCTCAGTAACAGTCCG 3′

*Alpl *	96	61.3°C	Forward: 5′ GGCCAGCTACACCACAACA 3′
		60.0°C	Reverse: 5′ CTGAGCGTTGGTGTTATATGTCTT 3′

*Bglap *	102	n.a.	Qiagen (QuantiTect Primer Assay KIT BGLAP)
			Cat. number: QT00259406
			(commercial product, no sequence available)

*Fmod *	145	62.7°C	Forward: 5′ AGCAGTCCACCTACTACGACC 3′
		62.2°C	Reverse: 5′ CAGTCGCATTCTTGGGGACA 3′

*Hprt *	173	67.1°C	Forward: 5′ GAGGAGTCCTGTTGATGTTGCCAG 3′
		66.4°C	Reverse: 5′ GGCTGGCCTATAGGCTCATAGTGC 3′

*Runx2 *	207	60.3°C	Forward: 5′ CCAACCGAGTCATTTAAGGCT 3′
		60.8°C	Reverse: 5′ GCTCACGTCGCTCATCTTG 3′
